# Arming oncolytic Coxsackievirus B3 with Neoleukin-2/15 enhances growth inhibition of colorectal carcinomas and induces T cell activation

**DOI:** 10.1016/j.omton.2026.201292

**Published:** 2026-07-14

**Authors:** Leslie Elsner, Maxim Girod, Ahmet Hazini, Anja Geisler, Lisanne Heimann, Babette Dieringer, Jackson Shipman-Mueller, Andreas Thiel, Robert Klopfleisch, Jens Kurreck, Sophie Van Linthout, Henry Fechner

**Affiliations:** 1 Technische Universität Berlin, Department of Applied Biochemistry, Institute of Biotechnology, Berlin, Germany; 2 Si-M “Der Simulierte Mensch”, a Science Framework of Technische Universität Berlin and Charité – Universitätsmedizin Berlin, Berlin, Germany; 3 University of Oxford, Department of Oncology, Old Road Campus Research Building (ORCRB), Roosevelt Drive, OX3 7DQ Oxford, UK; 4 Freie Universität Berlin, Department of Veterinary Medicine, Institute of Veterinary Pathology, Berlin, Germany; 5 Berlin Institute of Health (BIH) at Charité – Universitätsmedizin Berlin, BIH Center for Regenerative Therapies (BCRT), Berlin, Germany; 6 German Center for Cardiovascular Research (DZHK), Partner Site Berlin, Berlin, Germany; 7 Deutsches Herzzentrum der Charité (DHZC), Clinic of Cardiology, Angiology and Intensive Medicine, Campus Virchow Klinikum, Berlin, Germany

**Keywords:** cancer, colorectal carcinoma, immunotherapy, oncolytic virus, Coxsackievirus B3, PD-H, Neoleukin 2/15

## Abstract

Oncolytic viruses (OVs) represent an emerging class of cancer immunotherapeutics. Arming OVs with immunomodulatory transgenes is a promising strategy to reshape the tumor microenvironment (TME) and enhance therapeutic efficacy. We developed the oncolytic Coxsackievirus B3 (CVB3) strain PD-H, which has previously shown antitumor activity in colorectal and pancreatic cancer models. Here, we evaluated the capacity of PD-H to express transgenes and investigated whether its antitumor efficacy in colorectal cancer can be enhanced by insertion of the interleukin-2 (IL-2) mimetic Neoleukin-2/15 (Neo-2/15). Transgene insertion was best tolerated at the VP1-2A junction of the PD-H polyprotein, with viral replication and cytotoxicity inversely correlating with insert size. Genetic stability depended on the host cell line, transgene sequence and transgene length, with inserts up to 350 bp remaining stable for at least 10 viral passages. PD-H-derived Neo-2/15 was biologically active and induced proliferation of human CD4^+^ and CD8^+^ T cells *in vitro*. *In vivo*, intratumoral administration of PD-Neo-2/15 reduced the growth of subcutaneous Colon-26 tumors more effectively than PD-H and was associated with modulation of the TME, including an increased proportion of CD8^+^ T cells. Taken together, we demonstrate that arming PD-H with Neo-2/15 is a promising strategy to further enhance its anticancer efficacy.

## Graphical abstract

**Figure:**
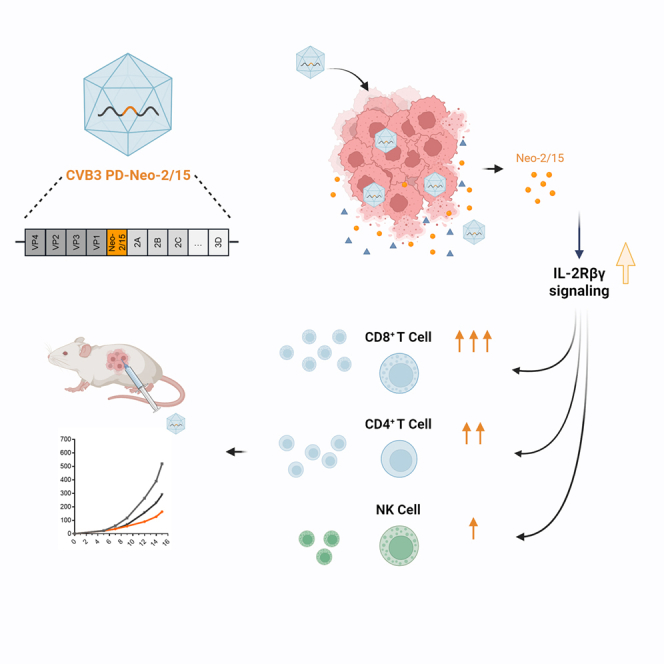

## Teaser

Oncolytic Coxsackievirus B3 strain PD-H was armed with the IL-2 mimetic Neoleukin-2/15 (Neo-2/15) to enhance antitumor efficacy in colorectal cancer. PD-Neo-2/15 expressed biologically active Neo-2/15, induced T cell proliferation *in vitro*, and significantly inhibited tumor growth *in vivo* while remodeling the tumor microenvironment toward an activated immune state.

## Introduction

Oncolytic viruses (OVs) are naturally occurring or engineered viruses that selectively replicate in tumor cells without harming normal cells. The antitumor efficacy of OVs results from two different closely linked mechanisms. One of these mechanisms is the direct virus-induced tumor cell lysis. Consequently, pathogen-associated molecular patterns (PAMPs), danger-associated molecular patterns (DAMPs), tumor-associated antigens, and neoantigens are released, thereby eliciting a subsequent antitumor immune response.^1^ Thus OVs activate antitumor immunity and convert immunologically “cold” tumors into “hot” ones, thereby overcoming a key mechanism of immune evasion that protects the tumor from immune attack. Importantly, the antitumor immunity is directed not only against OV-infected tumors but also against distant, non-infected metastasis, indicating that treatment with OVs induces systemic antitumor immunity.^2^^,^^3^

Coxsackievirus B3 (CVB3) is an OV whose oncolytic activity was described by Miyamoto et al. in 2012.^4^ The virus belongs to the picornavirus family. It has a positive-sense RNA genome with a length of approximately 7.5 kilobase pairs, which encodes for a single large open reading frame (ORF). The ORF is flanked by a 5′ untranslated region (5′ UTR), which contains an about 750 bp long internal ribosomal entry site, and a polyadenylated 3′ UTR of approximately 100 nt.^5^ The ORF encodes a polyprotein, which is processed into the 4 structural proteins (VP1-VP4) forming the viral capsid and 7 non-structural proteins (2A-2C, 3A-3D), as well as 3 intermediate cleavage products (2BC, 3AB, and 3CD), which are involved in virus replication.^5^^,^^6^ Processing of the polyprotein is mediated by two key cysteine proteases 2A^pro^ and 3C^pro^. The 2A^pro^ mediates a *cis*-autoproteolytic cleavage at the P1-P2 junction, thereby separating the structural P1 region from the nonstructural P2 and P3 regions. The majority of subsequent polyprotein processing events are carried out by the viral protease 3C^pro^ and its precursor 3CD, which together cleave the nonstructural regions into intermediate and mature.^7^^,^^8^

In recent years, potent antitumor activity of oncolytic CVB3 has been demonstrated *in vivo* in lung, breast, colon, pancreatic, and cervical squamous cell carcinomas.^9^^,^^10^^,^^11^^,^^12^ Several oncolytic CVB3 strains have been investigated for their antitumor efficiency, including CVB3 strain PD-H, which was initially derived from the CVB3 strain Nancy through adaption to human fibroblasts,^13^ and subsequently further adapted by our group to the viral producer cell line Chinese hamster ovary (CHO-K1).^9^ The virus has a unique receptor tropism, infecting tumor cells through binding to N- and 6-*O*-heparansulfates.^9^ Several studies have shown that PD-H efficiently inhibits the growth of colorectal and pancreatic tumors *in vivo* and prolongs animal survival. However, no complete cures were observed.^9^^,^^10^^,^^14^

Interleukin-2 (IL-2) is a potent cytokine that promotes T cell proliferation, the generation of cytotoxic CD8^+^ T cells, and the activation and persistence of natural killer (NK) cells. Owing to these immunostimulatory properties, recombinant IL-2 was the first immunotherapy approved for the treatment of melanoma and renal cell carcinoma.^15^ The biological activity of IL-2 is mediated through binding to the IL-2 receptor (IL-2R), which comprises the membrane-bound β (IL-2Rβ) and γ (IL-2Rγ) subunits.^16^^,^^17^ A third receptor component, IL-2Rα (CD25), does not directly participate in signal transduction but increases the affinity of IL-2 binding to the receptor complex.^17^^,^^18^^,^^19^ Interaction with the IL-2Rα subunit is detrimental in cancer therapy because the IL-2Rα subunit is expressed on off-target cells, including endothelial cells and immunosuppressive regulatory T cells (Tregs). These cells respond more strongly to high-dose IL-2 than intended target cells such as CD8^+^ naive T cells. Activated Tregs directly inhibit antitumor immunity by suppressing immune responses within the tumor microenvironment (TME)^20^ whereas interaction of IL-2 with IL-2Rαβγ on endothelial cells, contributes to the development of vascular leak syndrome (VLS), a severe side effect occurring as result of high-dose IL-2 treatment.^21^ Experimentally, VLS could be abrogated by a blocking antibody to IL-2Rα or its genetic disruption, indicating the importance of IL-2Rα for inducing VLS.^21^ Neoleukin-2/15 (Neo-2/15) is a *de novo* designed, heat- and denaturation-stable IL-2 mimetic that shares only 14% amino acid sequence homology with human IL-2.^22^ Unlike native IL-2, Neo-2/15 does not bind IL-2Rα but selectively engages the IL-2Rβγ complex, exhibiting a higher binding affinity than IL-2 while maintaining efficient downstream signal transduction. As a result, Neo-2/15 preferentially stimulates CD8^+^ T cells and NK cells while minimizing the activation of immunosuppressive Tregs, thereby enhancing antitumor immune responses.^22^^,^^23^ As a recombinant protein, Neo-2/15 has demonstrated significantly greater antitumor efficacy than recombinant IL-2 in syngeneic murine models of melanoma and colon cancer.^19^^,^^22^ Although there are no studies specifically investigating VLS, reduced treatment-associated toxicity with Neo-2/15 compared to IL-2 has been documented.^22^^,^^24^

In this study, we conducted a systematic investigation of the feasibility of inserting transgenes into PD-H. Based on these results, we assessed whether PD-H-mediated expression of Neo-2/15 enhances antitumor efficacy of the virus in colorectal cancer. Our data identify the VP1-2A junction within the PD-H polyprotein as the most suitable insertion site, allowing successful integration of foreign sequences up to 1,071 bp. Viral replication, cytotoxicity, and stability decreased with increasing transgene length, but were also influenced by transgene sequence and the host cell. Neo-2/15 was successfully expressed as functionally active protein from the engineered PD-H variant PD-Neo-2/15, inducing IL-2 receptor signaling and proliferation of CD4^+^ and CD8^+^ T cells *in vitro*. *In vivo*, PD-Neo-2/15 inhibited Colon-26 tumor growth more effectively than PD-H and was associated with alterations of the TME.

## Results

### Selection of transgene insertion sites and 2A and 3C protease CSs within the genome of PD-H

**Figure 1:**
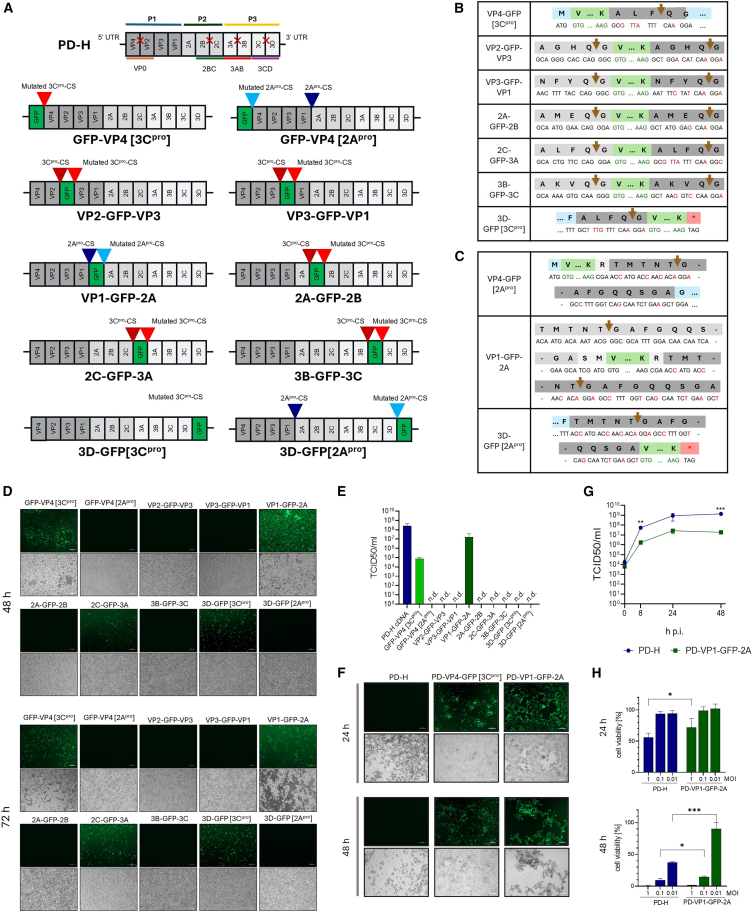
Systematic evaluation of transgene insertion sites in the CVB3 PD-H genome (A) Schematic overview of GFP transgene insertion sites in the PD-H genome. The PD-H genome is shown with red crosses indicating regions unsuitable for transgene insertion due to constraints of the viral replication cycle. Below, all tested insertion sites are depicted, ranging from upstream of VP4 to downstream of the 3D polymerase, including the respective protease CS. (B) Nucleotide and aa sequences of wild-type (light gray) and artificial (dark gray) 3C^pro^-CS with embedded GFP (green). Nucleotide substitutions introduced into the artificial CS are indicated in red. Viral aa are shown in light blue. The cleavage positions are indicated by brown arrows according to van Kuppeveld et al.^50^ (C) Nucleotide and aa sequences of wild-type (light gray) and artificial (dark gray) 2A^pro^-CS with embedded GFP (green). Nucleotide substitutions within the artificial CS are shown in red; viral aa are shown in light blue. The cleavage positions are indicated by brown arrows according to Höfling et al.^32^ (D) GFP expression (upper images) and cell lysis (lower images, light microscopy) after transfection of various PD-GFP cDNA constructs into HEK293T cells. Cells were transfected with 2.5 μg viral cDNA in six-well plates and analyzed 24 and 48 h later; Scale bars, 200 μm. (E) Viral titers after transfection of various PD-GFP cDNA constructs 72 h post-transfection, determined by TCID_50_ assay on HeLa cells; PD-H cDNA was used as control. Infectious virus was detected only for PD-H cDNA, GFP-VP4 [3C^pro^] and VP1-GFP-2A constructs; all other constructs failed to produce virus. (F) GFP expression (upper images) and virus-induced cell lysis (lower images, light microscopy) following infection with the viruses PD-H, PD-GFP-VP4 [3C^pro^], or PD-VP1-GFP-2A. HEK293T cells were infected with the viruses at MOI 0.01 and analyzed at 24 and 48 h post-infection (p.i.). Scale bars, 200 μm. (G) Virus growth kinetics. HEK293T cells were infected with PD-H or PD-VP1-GFP-2A at MOI 0.1. Viral titers were determined by TCID_50_ assay on HeLa cells at indicated time points; Significance: ∗∗*p* < 0.01, ∗∗∗*p* < 0.001. (H) Cell viability after infection with PD-H or PD-VP1-GFP-2A. HEK293T cells were infected with PD-H or PD-VP1-GFP-2A at the indicated MOIs. Cell viability was assessed by XTT assay at 24 and 48 h post-infection and normalized to untreated controls (100%); Significance: ∗*p* < 0.05, ∗∗∗*p* < 0.001. Data are presented as mean (SD) with *n* = 3, except for E, where *n* = 2 was used. The two-tailed unpaired *t* test was used for statistical analysis.

Given the critical role of sequential processing of the CVB3 polyprotein by the viral proteases 2A^pro^ and 3C^pro^ and the presence of naturally occurring 2A^pro^ and 3C^pro^ cleavage sites (CS) within the viral polyprotein,^25^ we identified eight possible sites for transgene insertion into the PD-H genome (Figure 1A). As a transgene, we selected the green fluorescent protein (GFP) reporter, as it is easily detectable and its 714 bp complementary DNA (cDNA) has been shown to be tolerated by other CVB3 strains.^26^ At three selected sites—the N terminus of VP4, the VP1-2A junction, and the C terminus of 3D—the transgene was inserted in fusion with an artificial 2A^pro^-CS. At seven sites—the N terminus of VP4, the VP2-VP3, VP3-VP1, 2A-2B, 2C-3A, and 3B-3C junctions, as well as the C terminus of 3D—the transgene was inserted in fusion with an artificial 3C^pro^-CS. The nucleotide sequences encoding the artificial 2A^pro^-CS and 3C^pro^-CS were engineered by synonymous nucleotide substitutions to minimize sequence homology with the native CS, thereby reducing the risk of transgene loss via homologous recombination (Figures 1B and 1C).^27^ To elucidate whether the transgene-containing PD-H cDNA constructs were able to generate viruses that express GFP, we transfected HEK293T cells with the 10 viral cDNAs constructs and analyzed the expression of GFP by fluorescence microscopy and virus generation by determining the TCID_50_. GFP was only detected in cells transfected with the constructs GFP-VP4 [3C^pro^], VP1-GFP-2A, 2C-GFP-3A, and 3D-GFP [3C^pro^] 48 and 72 h after transfection. Cells transfected with VP1-GFP-2A exhibited substantially stronger GFP expression than cells transfected with the other three constructs. First signs of cytolysis became evident 48 h after transfection but exclusively in cells transfected with the constructs GFP-VP4 [3Cpro] and VP1-GFP-2A. A pronounced cytolysis was detected in these cells 72 h after transfection, while none of the cells transfected with the other cDNA constructs exhibited cytolysis at this time point (Figure 1D). Accordingly, replicating virus was detected exclusively in GFP-VP4 [3C^pro^] and VP1-GFP-2A transfected cells, whereas virus detection failed in all other constructs (Figure 1E). The VP4-GFP [3C^pro^]-virus reached a titer of 3 × 10^4^, the VP1-GFP-2A-virus of 1 × 10^7^ TCID_50_/mL, while the parental PD-H virus without a transgene achieved 2 × 10^8^ TCID_50_/mL, indicating that the VP1-GFP-2A-virus replicated more efficiently than the GFP-VP4 [3C^pro^]-virus, though both were attenuated compared to PD-H. Next, we infected HEK293T cells with PD-H and the generated viruses PD-GFP-VP4 [3C^pro^] and PD-VP1-GFP-2A to confirm GFP expression and cytotoxicity. Both parameters were consistent with the cDNA transfection data, with PD-VP1-GFP-2A showing pronounced cytolysis comparable to PD-H, while PD-VP4-GFP [3Cpro] displayed only isolated plaques (Figure 1F). Based on these data, the VP1-2A junction was selected as the most suitable site for transgene insertion in PD-H. To directly compare the replication of PD-VP1-GFP-2A with PD-H, a one-step growth curve was conducted with both viruses at an MOI of 0.1. PD-VP1-GFP-2A replicated to titers of 10^7^ TCID_50_/mL at 48 and 72 h post-infection. Compared to PD-H, this was approximately 2 log_10_ lower (Figure 1G). A similar trend was observed for the cytotoxicity, which was determined by XTT-assay. Although PD-VP1-GFP-2A induced strong cytolysis, it remained below that of PD-H (Figure 1H).

These data demonstrate that PD-H tolerates a transgene when it is inserted N-terminal to the VP4-protein with a 3C^pro^-CS or at the VP1-2A junction, with the latter insertion site having less negative impact on viral performance in the producer cell line HEK293T.

### Transgene size is negatively correlated to generation of PD-H following cDNA transfection and increase of transgene length reduces stability of engineered PD-H viruses

**Figure 2:**
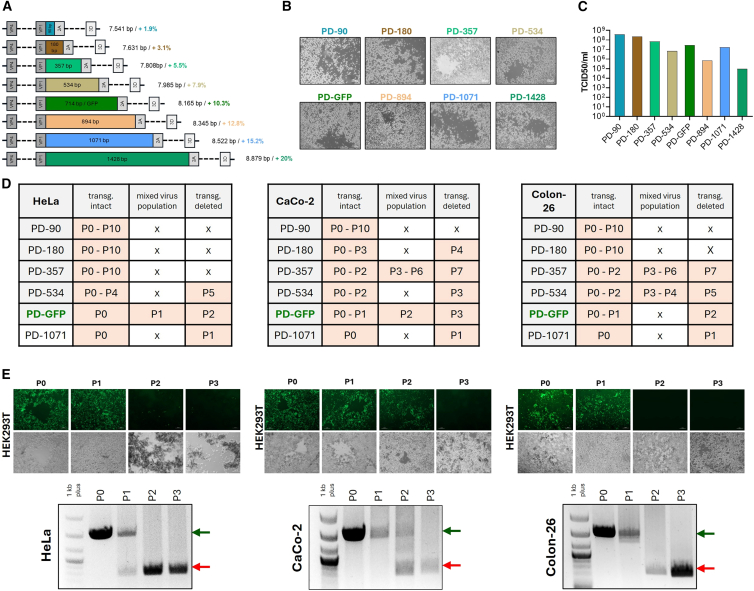
Transgene size is negatively correlated with PD-H generation and influences stability (A) Schematic overview of PD-H constructs carrying transgenes of varying sizes. Shorter inserts (90, 180, 357, and 534 bp) were generated by 3′ truncation of the GFP cDNA, whereas longer inserts (894, 1,071, and 1,428 bp) were generated using 3′-truncated luciferase cDNAs. The construct PD-GFP contains the GFP cDNA (714 bp). The total viral genome sizes of each construct and percentage increase of the genome compared to PD-H are indicated. Transgenes are flanked at the N terminus with a 2A^pro^-CS and at the C terminus end with a mutated 2A^pro^-CS. (B) Assessment of virus-induced cell lysis 72 h post-transfection of HEK293T cells with 2.5 μg DNA in a six-well plate. Treatment with all constructs resulted in cell lysis, as documented by light microscopy. (C) Viral titers measured 72 h post-transfection by TCID_50_ assay on HeLa cells. (D) Stability of engineered viruses up to P10. HeLa (left), CaCo-2 (middle), Colon-26 (right) cells were infected with PD-90, PD-180, PD-357, PD-534, PD-GFP, or PD-1071 at MOI 0.1. Virus-containing supernatants were transferred every 48 h to fresh cells at the same MOI. This process was repeated to P10. Viral RNA was extracted at each passage, reverse-transcribed, and PCR-amplified using primers flanking the foreign sequence. The table summarizes the passages with an intact transgene, passages showing mixed virus populations, and the passage at which transgene loss was observed. Transg., Transgene. (E) Stability of PD-GFP. HeLa (left), CaCo-2 (middle), Colon-26 (right) cells were infected with 0.1 MOI PD-GFP. Forty eight h later, the virus was harvested titrated and used to infect HEK293T cell with MOI 0.001. GFP expression (upper cell image) and cell viability (lower cell image) were determined by fluorescence and light microscopy 48 h post-infection, respectively. Lower gel images show PCR analysis of the PD-GFP genome from HeLa, CaCo-2, and Colon-26 cells at passages P0-P3, confirming presence or absence of the GFP-encoding sequence. Green arrow, PCR fragments with full-length transgene; red arrow, PCR fragments without transgene. Marker, 1 kb plus.

To investigate how transgene size affects virus generation, we inserted foreign sequences ranging in their size from 90 to 1,428 bp into the VP1-2A junction of PD-H genome. The smallest insert (90 bp) reflects the typical size of microRNA target sites, which are inserted into several oncolytic CVB3 to inhibit undesirable replication of the virus in normal tissues.^11^^,^^14^^,^^28^^,^^29^ Inserts in the 180–600 bp range represent small cytokines such as GM-CSF (435 bp), CXCL9 (372 bp), IL-2 (462 bp), and IFN-β (582 bp), which may be used to enhance antitumor immunity, whereas GFP (∼714 bp) can be used to simplify detection of gene expression. Inserts above ∼800 bp approach or exceed the size of more complex proteins. For example, the IL-12 p35 and p40 subunits, which have often been used to arm OVs,^30^^,^^31^ span approximately 1.1–1.4 kb. In our constructs, the transgenes from 90 to 534 bp encode for C-terminal truncated versions of GFP, whereas transgenes with a length of 894–1428 bp encode for C-terminal truncated versions of luciferase. Furthermore, the construct VP1-GFP-2A was also included (hereafter referred to as PD-GFP for simplicity) (Figure 2A). Following transfection of respective cDNAs all eight constructs induced cell lysis in HEK293T cells (Figure 2B) and led to generation of virus 72 h after transfection (Figure 2C). However, the virus titer progressively decreased with the increasing length of the transgenes inserted into the virus. The highest virus titer of 3.95 × 10^8^ TCID_50_/mL was determined for the virus PD-90 containing the smallest foreign gene sequence, whereas the lowest titer with up to 9.37 × 10^4^ TCID_50_/mL was found for the virus PD-1428, which contained the largest foreign sequence (Figure 2C). The data demonstrate that PD-H with foreign sequences up to 1,428 bp in length can be produced by transfecting viral cDNAs into HEK293T cells. However, our findings indicate that the efficiency of virus generation decreases distinctly as the size of the foreign sequence increases.

The stability of the transgene within the viral genome is crucial, as it determines both the strength and duration of its expression and is therefore a key factor in the oncolytic activity of an armed OV. Only six of the eight generated viruses were included in the investigation, as we were unable to amplify PD-894 and PD-1428 to titers sufficient to carry out the experiments. To assess the stability of the viruses PD-90 to PD-1071, we serially passaged them in HeLa cells, and colorectal carcinoma cell lines CaCo-2 and Colon-26 for a total of 10 passages (Figure 2D). After each passage, genomic viral RNA was isolated, and the foreign sequence was amplified using primers targeting the viral genome regions flanking the inserted foreign sequence. The resulting PCR fragments were then analyzed by agarose gel electrophoresis. Additionally, we also determined GFP expression of PD-GFP for passage (P) P0-P3 by fluorescence microscopy (Figure 2E). Analysis of viruses passaged in HeLa cells showed that PD-90, PD-180, and PD-357 remained stable up to passage 10 (P10). PD-534 lost its transgene at P5, while a portion of PD-GFP lost the GFP sequence as early as P1, though the GFP sequence was eliminated from the viral population in P2. The largest virus, PD-1071, lost its transgene the earliest, in P1. In CaCo-2 cells stable presence of the transgene to P10 was only detected for PD-90. PD-180 lost its foreign sequence at P4, PD-357 at P7, PD-534, PD-GFP at P3, and PD-1071 already at P1. In two viruses PD-357 and PD-GFP a mixed virus population at P3-P6 and at P2-containing viruses with and without foreign sequences was detected, respectively. In Colon-26 cells only PD-90 and PD-180 were stable up to P10, whereas PD-357 lost its transgene at P7, PD-534 at P5, PD-GFP at P2, and PD-1071 at P1. Mixed virus populations with and without transgene were found for PD-357 (P3 to P6) and PD-534 (P3 and P4) (Figure 2D).

These data demonstrate that the stability of the virus strongly depends on the size of the transgene. They also show that there are transitional states in which mixed populations of viruses with and without the transgenes coexist and that virus stability in addition to the length of the transgene is also influenced by the cell line used.

### Increase in transgene length impairs replication and cytotoxicity of the engineered PD-H

**Figure 3:**
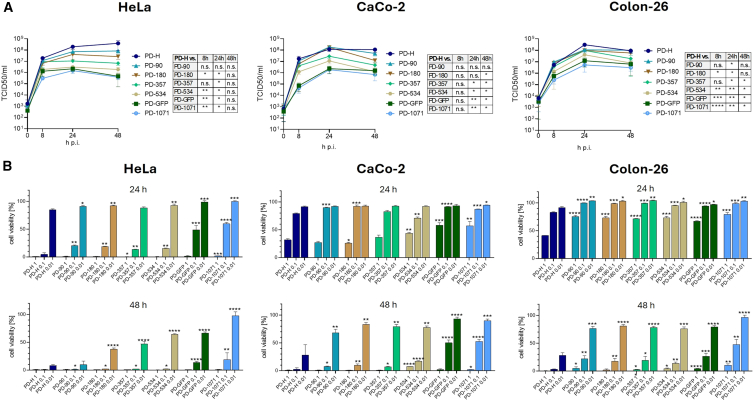
Increasing transgene size negatively affects viral growth and cytotoxicity (A) Virus replication. HeLa, CaCo-2 and Colon-26 cells were infected with PD-H, PD-90, PD-180, PD-357, PD-534, PD-GFP, and PD-1071 at MOI 0.1. Viral titers were determined by TCID_50_ assay on HeLa cells; Significance: ∗*p* < 0.05, ∗∗*p* < 0.01, ∗∗∗*p* < 0.001, ∗∗∗∗*p* < 0.0001; n.s., not significant. (B) Viral cytotoxicity. HeLa, CaCo-2, and Colon-26 cells were infected with the indicated viruses and MOIs. Cell viability was assessed by XTT assay at 24 and 48 h post-infection and normalized to untreated controls (100%). Comparisons were performed between PD-H carrying a transgene and parental PD-H at each MOI; Significance: ∗*p* < 0.05, ∗∗*p* < 0.01, ∗∗∗*p* < 0.001, ∗∗∗∗*p* < 0.0001. Data are shown as mean (SD) (*n* = 3). The two-tailed unpaired *t* test was used for statistical analysis.

To investigate how insertion of the transgenes affects the replication and lysis of PD-H, we next infected HeLa, CaCo-2, and Colon-26 cells with the engineered transgene containing PD-H constructs. In all three cell lines, the virus growth curves showed that replication gradually decreased as the size of the inserted foreign sequence increased. However, the strength of the effect depended on the cell lines studied. Thus, the virus PD-1071 replicated worst in all three cell lines, with HeLa cells showing a 1,000-fold poorer replication than PD-H, while in CaCo-2 and Colon-26 cells replication was only about 100-fold lower than that of PD-H. Simultaneously, viruses with foreign sequences up to a length of 180 bp replicated in HeLa cells only about 10-fold worse than PD-H, whereas in CaCo-2 and Colon-26 cells a similar reduction in viral replication was observed for viruses containing foreign sequences of up to 357 and 534 bp, respectively (Figure 3A). The cytotoxicity of the viruses also decreased with increasing size of the foreign sequence, and the insertion of as little as 90 bp of foreign sequences already resulted in a significant reduction in cytotoxicity in all cell lines compared to PD-H. However, HeLa cells were overall markedly more sensitive to the viruses than CaCo-2 and Colon-26 cells (Figure 3B).

In summary, these data demonstrate that the insertion of a foreign sequence into PD-H inhibits viral replication and cytotoxicity. Moreover, the magnitude of the reduction is inversely proportional to the size of the foreign sequences and is influenced by the host cell line.

### Generation and characterization of PD-Neo-2/15 and PD-Neo-2/15-His

**Figure 4:**
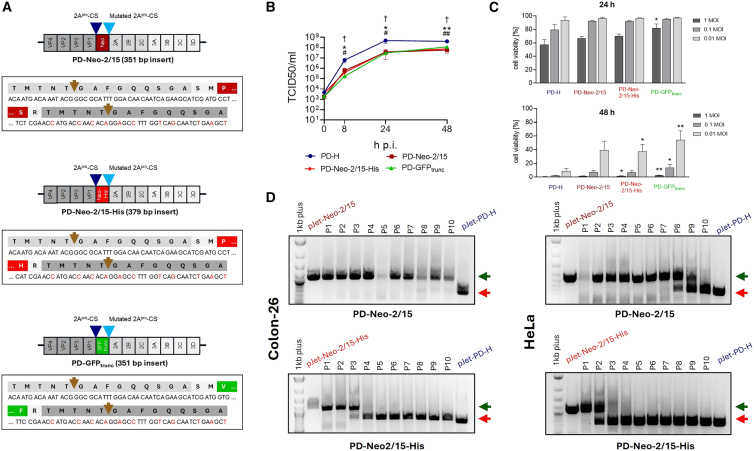
Characterization of PD-Neo-2/15 and PD-Neo-2/15-His (A) Schematic representation of PD-Neo-2/15, PD-Neo-2/15-His and PD-GFP_trunc_ constructs. Nucleotide and amino acid sequences of native (light gray) and artificial (dark gray) 2A^pro^-CS with embedded transgenes (dark red, light red, green). Nucleotide substitutions within the sequence encoding for the artificial 2A^pro^-CS are shown as red letters. Cleavage positions are indicated by brown arrows according to van Kuppeveld et al.^50^ (B) Virus growth kinetics in Colon-26 cells. Cells were infected with PD-H, PD-Neo-2/15, PD-Neo-2/15-His or PD-GFP_trunc_ at MOI 0.1. Virus titers were measured at indicated time points using the TCID_50_ method on HeLa cells. Significance: PD-H vs. PD-Neo-2/15: ∗*p* < 0.05, ∗∗*p* < 0.01; PD-H vs. PD-Neo-2/15-His: ^#^*p* < 0.05, ^##^*p* < 0.01; PD-H vs. PD-GFP_trunc_: ^†^*p* < 0.05. (C) Cell viability of Colon-26 cells following infection. Cells were infected with PD-H, PD-Neo-2/15, PD-Neo-2/15-His, or PD-GFP_trunc_ at the indicated MOIs. Viability was assessed by XTT assay at 24 h (top) and 48 h (bottom) post-infection and normalized to untreated controls (100%); Significance PD with transgene vs. PD-H: ∗*p* < 0.05, ∗∗*p* < 0.01. (D) Transgene stability in Colon-26 and HeLa cells. Cells were infected with PD-Neo-2/15 or PD-Neo-2/15-His at MOI 0.1 and harvested after 48 h. Virus-containing supernatants were serially passaged on the respective cell line at MOI 0.1 for up to 10 passages. Viral RNA was extracted from each passage, reverse-transcribed, and PCR-amplified using primers flanking the Neo-2/15 or Neo-2/15-His insert. PCR products were analyzed by 1% agarose gel electrophoresis. Green arrows, PCR fragments with full length transgene; red arrows, PCR fragments without transgene. pJet-Neo-2/15, pJet-Neo-2/15-His and pJet-PD-H represent plasmids containing the cDNAs of PD-Neo-2/15, PD-Neo-2/15-His and PD-H, respectively. Data are shown as mean (SD) (*n* = 3). The two-tailed unpaired *t* test was used for statistical analysis.

To analyze whether arming of PD-H with an immunomodulatory transgene can improve its oncolytic activity in colorectal cancer, we next inserted the cDNA of Neo-2/15 (length 351 bp, Neo-2/15) or a cDNA of a His-tag-extended Neo-2/15 (length 379 bp) into the VP1-2A junction of PD-H and generated the viruses PD-Neo-2/15 and PD-Neo-2/15-His. We also generated the control virus, PD-GFP_trunc_, containing a truncated GFP cDNA, which has the same length as the cDNA of Neo-2/15 (Figure 4A). The murine cDNA of IL-2 was also cloned into the VP1-2A junction of PD-H. However, for unknown reasons, we were unable to generate the respective virus after transfection of the cDNA plasmid into the HEK-293T producer cell line. Growth kinetics of the viruses PD-Neo-2/15, PD-Neo-2/15-His, and PD-GFP_trunc_ were determined in Colon-26 cells following infection with MOI 0.1. PD-Neo-2/15 and PD-Neo-2/15-His reached similar titers ranging between 5 × 10^7^ and 8 × 10^7^ TCID_50_/mL. The titers of PD-GFP_trunc_ reached 1 × 10^8^ TCID_50_/mL. These values however, were up to 10-fold lower than those of PD-H (Figure 4B). The three engineered viruses also exhibited similar and strong cytotoxicity after infection of Colon-26 cells with 0.01–1 MOI, yet they did not fully reach the level of cytotoxicity induced by PD-H (Figure 4C). To assess the genetic stability of PD-Neo-2/15 and PD-Neo-2/15-His, both viruses were serially passaged in Colon-26 and HeLa cells. The coding sequence of Neo-2/15 within the PD-H genome was detected in Colon-26 cells up to P10. Between P8 and P10, a mixed viral population was observed, consisting of a low proportion of variants lacking the transgene and a high proportion retaining it. In contrast, in HeLa cells the transgene was lost at P10 and a mixed virus population containing the coding sequence of Neo-2/15 was detected at P8 and P9. PD-Neo-2/15-His showed distinctly lower stability than PD-Neo-2/15. In both Colon-26 and HeLa cells, a mixed virus population with and without transgene became already visible at P3 (Colon-26) and P2 (HeLa). The coding sequence of Neo-2/15-His was lost in Colon-26 cells at P4 and in HeLa cells at P3, respectively (Figure 4D).

These results show that PD-H can be equipped with Neo-2/15 and Neo-2/15-His. However, in terms of replication and cytotoxicity both viruses exhibit weaker performance than PD-H. Regarding virus stability, PD-Neo-2/15 was clearly superior to PD-Neo-2/15-His. Consequently, only PD-Neo-2/15 was pursued in subsequent investigations.

### Neo-2/15 expressed from PD-H enables activation of IL-2 receptor signaling and induces proliferation of human T cells *in vitro*

**Figure 5:**
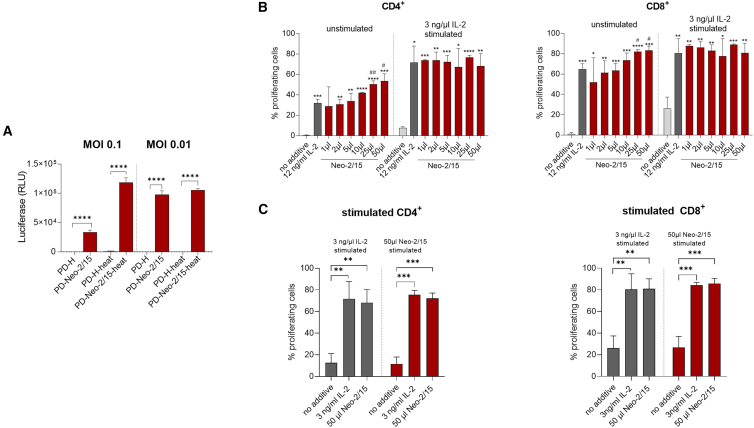
Induction of T cell proliferation by Neo-2/15 (A) IL-2Rβγ bioassay confirming Neo-2/15 functionality. HEK293T cells were infected with PD-H or PD-Neo-2/15 at MOI 0.1 or 0.01. After 48 h, supernatants were collected, centrifuged, and analyzed in an IL-2Rβγ bioassay. Heat-inactivated samples (30 min, 60°C) retained activity, consistent with the heat stability of Neo-2/15; Significance: ∗∗∗∗*p* < 0.0001. (B) Proliferation of CD4^+^ and CD8^+^ T cells in response to IL-2 or Neo-2/15. Cells were stimulated with anti-CD3 and anti-CD28 or left unstimulated for 48 h and subsequently cultured for 5 days with no additive (control), 12 ng/mL recombinant IL-2, or indicated volumes of heat-inactivated viral supernatant containing Neo-2/15. Supernatant was derived from HEK293T-cells infected with PD-Neo-2/15 at MOI 0.1 for 48 h. Proliferation was assessed by CFSE dilution and flow cytometry; Significance: ∗*p* < 0.05, ∗∗*p* < 0.01, ∗∗∗*p* < 0.001, ∗∗∗∗*p* < 0.0001 (untreated vs. treated); ^#^*p* < 0.05, ^##^*p* < 0.01 (IL-2 vs. Neo-2/15). (C) Proliferation of stimulated CD4^+^ and CD8^+^ T cells in response to Neo-2/15. Cells were stimulated with anti-CD3 and anti-CD28 in the presence of 50 μL heat-inactivated viral supernatant containing Neo-2/15 or 3 ng/mL IL-2 (control) for 48 h and subsequently cultured for 5 days with the same respective stimuli. Proliferation was assessed by CFSE dilution and flow cytometry; Significance: ∗∗*p* < 0.01, ∗∗∗*p* < 0.001. Data are shown as mean (SD) (*n* = 3). The two-tailed unpaired *t* test was used for statistical analysis.

To confirm the functionality of PD-H-expressed Neo-2/15, we investigated whether the protein induces IL-2R signaling. HEK293T cells were infected with MOI 0.1 and 0.01 MOI of PD-H and PD-Neo-2/15. The cell culture supernatant was collected 48 h later, centrifuged, and used natively or after virus inactivation by heating in an IL-2Rβγ bioassay. Both native and heat-treated supernatants from PD-Neo-2/15, but not from PD-H-infected cells, strongly induced luciferase expression in this assay, demonstrating that Neo-2/15 was expressed as a functionally active protein from the virus capable of inducing IL-2Rβγ signaling (Figure 5A).

To assess whether IL-2Rβγ induction led to induction of human T cell proliferation, primary human T cells were stimulated using anti-CD3/anti-CD28 antibodies in the presence of 3 ng/μL recombinant IL-2 or were left unstimulated for 48 h. Thereafter, the cells were cultured for further 5 days in the presence of 12 ng/mL recombinant IL-2 or increasing volumes (1–50 μL) of heat-inactivated supernatant harvested from HEK293T cells after infection with 0.1 MOI PD-Neo-2/15 for 48 h. Cell proliferation was assessed by reduction in carboxyfluorescein diacetate succinimidyl ester (CFSE) signal and flow cytometry. In both, unstimulated and stimulated primary T cells, treatment with IL-2 and Neo-2/15 resulted in induction of CD4^+^ and CD8^+^ T cell proliferation. In unstimulated T cells induction of proliferation was distinctly lower than in stimulated cells and increased as more Neo-2/15-containing supernatant was used, whereas in the stimulated T cells increase of volume of Neo-2/15-containing cell culture supernatant did not further lead to increase of CD4^+^ and CD8^+^ T cell proliferation (Figure 5B). Interestingly, already 1–2 μL of Neo-2/15-containing supernatant was sufficient to achieve cell proliferation similar to that induced by 12 ng/mL IL-2, confirming the high activity of Neo-2/15. To elucidate, whether Neo-2/15, similar to IL-2, can provide the third stimulatory signal required for T cell proliferation during the initial 48-h activation with antiCD3/anti-CD28, we repeated the experiment by replacing recombinant IL-2 by Neo-2/15-containing supernatants. As shown in Figure 5C, virus expressed Neo-2/15 was able to fully replace the function of IL-2.

These data demonstrate that Neo-2/15 is expressed as functional protein from PD-Neo-2/15, which is able to induce IL-2Rβγ signaling and CD4^+^ and CD8^+^ proliferation.

### PD-Neo-2/15 reduces growth of s.c. Colon-26 tumors and leads to changes in the TME in Balb/C mice *in vivo*

**Figure 6:**
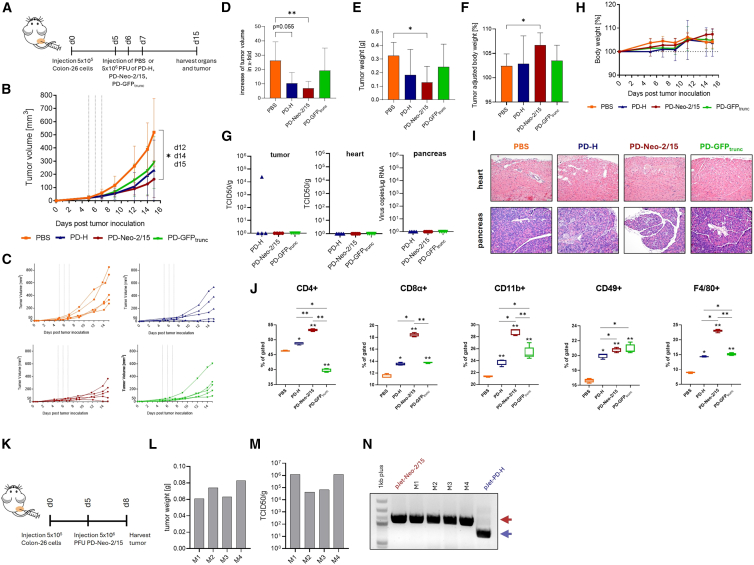
*In vivo* oncolytic efficacy and transgene stability of PD-Neo-2/15 in the Colon-26 syngeneic mouse tumor model (A) Experimental timeline. A total of 5 × 10^5^ Colon-26 cells were injected subcutaneously (s.c.) into the right flank of Balb/C mice. Five days later, tumors were treated intratumorally (i.t.) on three consecutive days (days 5–7) with 5 × 10^6^ PFU of PD-H, PD-Neo-2/15, PD-GFP_trunc_, or PBS (*n* = 5 per group). Tumors and organs were collected at the indicated time point. (B) Tumor growth curves. Dashed lines indicate virus injections. (C) Individual tumor volumes corresponding to (B), shown per animal. (D) Increase in tumor volume, calculated as the ratio of tumor volume at day 15 to day 5; Significance: ∗∗*p* < 0.01. (E) Tumor weights at experimental endpoint (day 15); Significance: ∗*p* < 0.05. (F) Tumor-adjusted body weight on day 15. Tumor mass was subtracted from total body weight and normalized to baseline (day 0); Significance: ∗*p* < 0.05. (G) Biodistribution of viruses in tumor, heart, and pancreas on day 15. Viral titers in tumor and heart were determined by TCID_50_ assay; viral genome copy numbers in pancreas were assessed by qRT-PCR. Data are shown per animal. (H) Percent change in total body weight over the 15-day experimental period. (I) Histopathological analysis of heart and pancreas tissues. Representative H&E-stained sections on day 15. (J) Flow cytometric analysis of tumor-infiltrating immune cells on day 15. Proportion of CD4^+^ T cells, CD8α^+^ T cells, CD11b^+^ myeloid cells, CD49^+^ NK cells, and F4/80^+^ macrophages are shown as percentages of viable CD45^+^ cells; *n* = 5 for PBS, PD-Neo-2/15, and PD-GFP_trunc_; *n* = 4 for PD-H; Significance: ∗*p* < 0.05, ∗∗*p* < 0.01 (K) Schematic representation of the experimental timeline for transgene stability assessment. (L) Tumor weight on day 8 for each animal. (M) Tumor titers of PD-Neo-2/15 on day 8, determined by TCID_50_ assay. (N) Transgene stability of PD-Neo-2/15 3 days after intratumoral injection. Viral RNA was extracted from tumor lysates, reverse-transcribed, and PCR-amplified using primers flanking the Neo-2/15 insert. PCR products were analyzed by 1% agarose gel electrophoresis. Green arrow, PCR fragments with full length transgene; red arrows, PCR fragments without transgene. pJet-Neo-2/15 and pJet-PD-H represent plasmids containing the cDNAs of PD-Neo-2/15 and PD-H, respectively. M1 to M4, mouse 1 to mouse 4. Data are shown as mean (SD). The Mann-Whitney *U* test was used for statistical analysis.

To assess whether arming PD-H with Neo-2/15 enhances its oncolytic activity, we established subcutaneous (s.c.) Colon-26 tumors in Balb/c mice. On day 5 after Colon-26 cell injection animals were randomly divided into four groups and intratumorally (i.t.) injected with 5 × 10^6^ plaque-forming unit (PFU) of PD-Neo-2/15, PD-H, or PD-GFP_trunc_ or PBS on three consecutive days. Tumor growth was monitored until day 15 after Colon-26 cell inoculation. The experiment was terminated at this point, because the skin over various tumors showed initial signs of ulceration, requiring euthanasia of the animals for welfare reasons (Figure 6A). Tumors in the PBS group grew rapidly and the average tumor volume reached 518.9 mm^3^ at the end of the experiment. All three virotherapy treatments delayed tumor growth compared with the untreated control group. The average tumor growth in the PD-Neo-2/15 group was significantly reduced between days 12 and 15, reaching a 68.4% (163.68 mm^3^) reduction by day 15. Tumor growth was also reduced in the PD-H group by 55.3% (231.66 mm^3^) and in the PD-GFP_trunc_ group by 44.2% (289.52 mm^3^), although these reductions did not reach statistical significance (Figure 6B). Furthermore, tumor shrinkage occurred in one animal in each of the PD-Neo-2/15 and PD-H groups (Figure 6C). As an additional measure of treatment efficiency, we determined the tumor volume after explantation. In the PBS group, tumor volume increased 26.1-fold, in the PD-GFP_trunc_ group 19.3-fold, in the PD-H group 10.2-fold, and in the PD-Neo-2/15 group only 6.8-fold from day 5 to day 15. In the PD-Neo-2/15 group, the reduction in tumor volume was statistically significant, whereas in the PD-H group, there was a clear trend toward significance (Figure 6D). We also measured the tumor weight after explantation at day 15. The highest tumor weights were observed in animals from the PBS control group, reaching an average tumor weight of 0.325 g, whereas in the PD-GFP_trunc_ group, the average tumor weight was 0.243 g, in the PD-H group 0.182 g and in the PD-Neo-2/15 group 0.127 g. A significant reduction of tumor weight was only observed for the PD-Neo-2/15 group (Figure 6E). Similar data were obtained when tumor weight was related to body weight. Also, here a significant reduction compared to PBS control was only observed for the PD-Neo-2/15 group (Figure 6F). Infectious virus particles were not recovered from tumor tissues at day 15, with the exception of a single animal treated with PD-H (Figure 6G), suggesting efficient viral clearance at this time point. Regarding treatment safety, no significant differences in body weight development were observed between the PBS control group and the virus-treated groups (Figure 6H). Moreover, no virus was detected in the pancreas or heart (Figure 6G), organs known to be particularly susceptible to CVB3 infection in mice, and no signs of inflammation, tissue damage, or tissue edema were observed in these tissues across all treatment groups after histological examination (Figure 6I), supporting the favorable safety profile of PD-Neo-2/15 and the other PD-H variants. To assess whether the enhanced antitumor efficacy of PD-Neo-2/15 is associated with changes in the TME, tumor-infiltrating immune cells from explanted tumors were analyzed using flow cytometry. Analysis of relative immune cell frequencies revealed significant increase in CD8^+^ T cells in PD-Neo-2/15-treated tumors compared to PBS controls (18.4% vs. 11.5%). Importantly, PD-Neo-2/15-treated tumors showed a significantly higher proportion of CD8^+^ T cells compared to PD-H and PD-GFP_trunc_ treatment, which only induced a modest increase over PBS-treated controls (13.5% and 13.8%, respectively). A similar effect was observed for CD4^+^ T cells, with PD-Neo-2/15 treatment leading to an increased frequency compared to PBS controls (53.3% vs. 46.2%). Moreover, PD-Neo-2/15 treatment resulted in a significantly higher proportion of CD4^+^ T cells in the tumor than treatment with PD-H (48.8%) or PD-GFP_trunc_ (39.75%). Analysis of myeloid populations demonstrated that PD-Neo-2/15-treated tumors exhibited significantly elevated proportions of CD11b^+^ myeloid cells (28.8% vs. 21.3%), CD49^+^ NK cells (20.8% vs. 16.6%), and F4/80^+^ macrophages (23.0% vs. 9.0%) relative to PBS control tumors. A significantly higher proportion of CD11b^+^ and F4/80^+^ cells was observed in PD-Neo-2/15-treated tumors compared to those treated with PD-H (23.8% and 14.4%, respectively) and PD-GFP_trunc_ (25.4% and 14.9%, respectively). In contrast, the proportion of CD49^+^ cells in PD-Neo-2/15-treated tumors was significantly increased compared to PD-H (20.0%), but not compared to PD-GFP_trunc_ (21.0%) (Figure 6J). To assess the stability of PD-Neo-2/15 in tumors, we conducted an additional experiment. Colon-26 tumors were established (s.c.) in four animals, treated with PD-Neo-2/15, and harvested 3 days post-virus infection (Figure 6K). At that time, tumors weighted between 0.06 and 0.08 g (Figure 6L). Infectious virus could be recovered from all infected tumors, with virus titers ranging between 4 × 10^4^ and 1 × 10^6^ TCID_50_/g (Figure 6M). To evaluate transgene stability, viral RNA was isolated from tumor tissue, reverse-transcribed, and subjected to PCR using primers flanking the Neo-2/15 transgene. PCR analysis revealed that the viral genomes of all four tumors harbored an intact Neo-2/15 insert (Figure 6N).

Taken together, these data indicate that PD-Neo-2/15 more effectively inhibits Colon-26 tumor growth than PD-H, without inducing detectable side effects, and is associated with pronounced modulation of the TME toward an activated immune state. Together, the detection of infectious virus in tumor tissue and the pronounced immune cell infiltration suggest that tumor cell elimination in PD-Neo-2/15-treated animals results from both direct virus-induced oncolysis and subsequent immune-mediated tumor cell killing. Furthermore, PD-Neo-2/15 remains genetically stable within tumor tissue for at least 3 days after administration.

## Discussion

The feasibility of transgene expression from OVs depends on multiple factors, of which viral genome size represents a major constraint. CVB3 possesses a comparatively small positive-strand RNA genome of approximately 7.5 kb, which inherently limits the size of foreign sequences that can be inserted.^32^^,^^33^ Unlike larger OV genomes such as adenoviruses or herpesviruses, CVB3 lack non-essential genes that could accommodate transgene insertions without interfering with viral coding regions. An additional challenge arises from the requirement to integrate transgenes into the viral polyprotein. Polyprotein processing in CVB3 is highly complex and tightly regulated, and transgene insertions can markedly impair viral replication or completely abrogate infectivity.^34^ Previous studies have demonstrated that CVB3 tolerates insertion of interleukins,^27^^,^^35^ small polypeptides,^32^^,^^36^ minigenes,^37^ or reporter genes such as GFP^26^^,^^38^^,^^39^ at specific sites of the polyprotein. However, a systematic analysis of permissive insertion sites and their impact on replication, cytotoxicity, and genomic stability of an oncolytic CVB3, as well as the potential of immunomodulatory transgenes to enhance its antitumor efficacy, has not been previously addressed. To address these limitations, we investigated which regions within the polyprotein of the oncolytic CVB3 PD-H are permissive for transgene insertion using GFP as a reporter. Among eight candidate sites, productive virus generation, and robust GFP expression were only observed when GFP was inserted at the VP1-2A junction or at the N terminus of the PD-H viral polyprotein. However, the VP1-2A junction was markedly more suitable for transgene insertion, as insertion of the transgene at this site supported higher levels of viral replication and GFP expression. In contrast, insertion at two other sites of the viral polyprotein resulted in detectable GFP expression but failed to support viral replication, indicating that polyprotein processing alone is insufficient to preserve viral fitness. Our data further demonstrate that the choice of the protease CS separating the transgene from viral proteins critically influences viral viability. While insertion of an artificial 3C^pro^ CS at the N terminus of the VP4 protein preserved viral replication, use of an artificial 2A^pro^-CS at this position was not tolerated, suggesting that even subtle alterations can compromise the virus.

To assess the impact of transgene size on viral performance, we inserted foreign sequences ranging from 90 to 1,428 nt at the VP1-2A junction. Although infectious virus could initially be generated from all constructs, increasing transgene length progressively impaired viral replication, cytotoxicity, and genomic stability. Very large inserts (>800 nt) severely compromised viral amplification and were unstable, whereas medium-sized transgenes (approximately 350–550 nt) best maintained replication and stability. Small inserts (<350 nt) were generally well tolerated. Interestingly, transgene stability was not determined solely by its size. In fact, PD-Neo-2/15 displayed greater genomic stability than a PD-H variant containing a His-tagged version of Neo-2/15, despite both constructs having a similar length. In particular, the repetitive histidine residues present in Neo-2/15-His may promote recombination events during viral minus-strand synthesis, a mechanism that has been implicated in transgene excision in picornaviruses.^40^ In addition, we observed that transgene stability is influenced by the cellular environment. This may be relevant for cancer treatment in certain patients, as early transgene loss could reduce therapeutic efficacy.

Arming OVs with immunostimulatory transgenes represents a widely used strategy to enhance their therapeutic efficacy. Successful approaches include the expression of immune checkpoint-targeting single-chain variable fragments (e.g., PD-1 or PD-L1)^41^^,^^42^ as well as pro-inflammatory cytokines (e.g., GM-CSF, IL-2, IL-12, IL-15) or chemokines (e.g., CCL2, CCL5, CCL19, CXCL11).^43^ Moreover, compared to systemic administration, OV-mediated expression of immunostimulatory proteins enables tumor-restricted enrichment of the proteins thereby enhancing efficacy while reducing systemic toxicity.^44^^,^^45^ Neo-2/15 exhibits strong immunomodulatory activity and, owing to its size (357 bp), stability within the viral genome, and ability to be expressed as a biologically active transgene, fully meets the requirements for expression by PD-H. Treatment of s.c. Colon-26 tumors with PD-Neo-2/15 resulted in significant tumor growth inhibition, which was more pronounced than that observed with PD-H. These findings confirm that arming with Neo-2/15 enhances the therapeutic efficacy of PD-H *in vivo*. Remarkably, enhanced antitumor activity was observed despite the reduced replication efficiency of PD-Neo-2/15 relative to PD-H, indicating that Neo-2/15-mediated immunostimulation can offset, and potentially exceed, the diminished intrinsic oncolytic activity associated with its insertion into the virus. Furthermore, no virus- or transgene-associated adverse effects were observed in treated animals. This finding reflects the favorable safety profile of PD-Neo-2/15, which likely results from the tumor-selective replication of PD-H and the selective activation of IL-2Rβγ signaling by Neo-2/15 without engagement of the IL-2Rα subunit, thereby limiting the toxicity associated with the natural IL-2 analog.^22^

Our findings on the size-dependent stability of the transgene within PD-H raise important questions regarding the long-term therapeutic efficacy achieved with PD-Neo-2/15. The Neo-2/15 encoding sequence within the viral genome remained detectable within the tumor for at least 3 days after PD-Neo-2/15 administration, whereas the Neo-2/15-induced remodeling of the TME was still evident 10 days after PD-Neo-2/15 injection. Together with the demonstrated *in vitro* stability of the transgene, these findings suggest sustained biological activity of virally expressed Neo-2/15 *in vivo*. However, the duration of therapeutic transgene activity is determined not only by transgene stability but also by progressive viral clearance mediated by the host immune response. Consistent with this notion, PD-Neo-2/15 was barely detectable in tumor tissue 10 days after virus administration, indicating that transgene expression had likewise largely ceased. To compensate for the loss of viral genomes and thereby prolong transgene expression, repeated administration of the virus represents a rational strategy. Indeed, repeated dosing is already an integral component of clinical oncolytic virotherapy protocols^46^ and may therefore also be an appropriate strategy for PD-Neo-2/15.

Consistent with the improved antitumor efficacy, PD-Neo-2/15 treatment was associated with distinct alterations of the TME to an activated immune status. In particular, tumors treated with PD-Neo-2/15 exhibited an increased i.t. proportion of CD8^+^ T cells, accompanied by moderate changes in CD4^+^ T cell frequencies, which both were significantly higher compared with those observed in tumors treated with the control viruses. CD8^+^ T cells represent central mediators of antitumor immunity by recognizing tumor-associated antigens presented on malignant cells and inducing direct tumor cell killing through cytotoxic effector mechanisms, including perforin/granzyme-mediated apoptosis and Fas/FasL interactions. In the context of oncolytic virotherapy, this adaptive immune response is initiated and amplified by virus-induced tumor cell lysis, which releases immunogenic signals that convert the TME from an immunosuppressed to an inflamed state.^47^ Accordingly, an increase of CD8^+^ T cells in the tumor tissue has been associated with improved patient survival across a wide range of solid malignancies.^48^ Furthermore, i.t. proportions of CD11b^+^ and F4/80^+^ macrophages were also significantly increased following PD-Neo-2/15 treatment, indicating modulation of myeloid cell compartments with stimulating activity within the TME. Our data are consistent with those of a recently published study by Wu et al.,^24^ who reported immune activation within the TME following treatment of colorectal MC38 tumors with an oncolytic VSV expressing Neo-2/15. Together, these findings identify Neo-2/15 as an immunomodulatory protein capable of enhancing the oncolytic activity of OVs. To further elucidate the mechanistic basis of the observed antitumor immune responses elicited by PD-Neo-2/15, future studies could incorporate a more comprehensive immune profiling of PD-Neo-2/15-treated tumors. Such analyses may include the assessment of T cell exhaustion markers, including PD-1, TIM-3, and LAG-3, detailed cytokine profiling, and functional *ex vivo* T cell assays to provide deeper insight into the underlying immune mechanisms.

In conclusion, this study provides a comprehensive characterization of an oncolytic CVB3 strain for accommodating and expressing a tumor-toxic transgene. Moreover, we demonstrate that the IL-2 analog Neo-2/15 can be expressed from PD-H as a biologically active, immunostimulatory protein that enhances antitumor efficacy of the parental virus, without adverse effects. Critically, the enhanced antitumor activity of PD-Neo-2/15 was accompanied by a pronounced remodeling of the TME, characterized by increased infiltration of CD4+ T cells, CD8^+^ T cells, CD49+ NK cells, CD11b^+^ myeloid cells, and F4/80^+^ macrophages, underscoring immune cell recruitment and activation as a major driver of therapeutic efficacy. Collectively, these findings establish PD-Neo-2/15 as a next-generation oncolytic CVB3 and provide a rational framework for future optimization and application of this OV platform. Future studies should evaluate PD-Neo-2/15 in orthotopic and metastatic colorectal cancer models to further assess its translational potential.

## Material and methods

### Cell culture

HEK293T cells were grown in high glucose Dulbecco's Modified Eagle Medium (DMEM) (Biowest, Darmstadt, Germany) with 10% fetal calf serum (FCS), 1% penicillin/streptomycin (P/S), 1% L-glutamine, and 1% sodium pyruvate. HeLa cells were maintained in Minimum Essential Medium (MEM) (Gibco, Karlsruhe, Germany) supplemented with 5% FCS, 0.02 M HEPES, 1% non-essential aas (NEAA), and 1% P/S. The murine colorectal cell line Colon-26 was grown in RPMI 1640 (c.c.pro, Oberdorla, Germany) supplemented with 10% FCS, 1% P/S, 1% L-glutamine, and 2% sodium pyruvate and the human colorectal cell line CaCo-2 was maintained in high glucose DMEM (Biowest) supplemented with 10% FCS, 1% P/S, 1% L-glutamine, 1% NEAA, and 1% sodium pyruvate.

### Cloning of GFP into cDNA of PD-H

The plasmid construct pJet-PD-VP1-GFP-2A was generated using the In-Fusion HD Cloning Kit (Takara Bio USA, Mountain View, CA, USA), with the GFP sequence containing a mutated 2A^pro^-CS downstream, synthesized via gene synthesis (Thermo Fisher Scientific, Waltham, MA, USA). The plasmid constructs pJet-PD-VP2-GFP-VP3, pJet-PD-VP3-GFP-VP1, pJet-PD-2A-GFP-2B, pJet-PD-2C-GFP-3A, and pJet-PD-3B-GFP-3C were assembled through multiple cloning steps. In the initial step, the GFP transgene was inserted along with a universal 3C^pro^-CS downstream. Subsequently, the nucleotide sequence of the 3C^pro^-CS was specifically modified using mutagenic primers to ensure that the resulting aa sequence matched the natural sequence found in the viral genome upstream of the GFP transgene, while the nucleotide sequence itself remained non-homologous. For the construct pJet-PD-3D-GFP [2A^pro^], the GFP sequence with an upstream mutated 2A^pro^-CS was synthesized via gene synthesis and the plasmid was assembled using the In-Fusion HD Cloning Kit (Takara Bio). For the generation of pJet-PD-3D-GFP [3C^pro^], the GFP transgene, including the 3C^pro^-CS from the pJet-PD-2C-GFP-3A construct, was amplified and assembled using the In-Fusion HD Cloning Kit (Takara Bio). Similarly, for the construction of pJet-PD-GFP-VP4 [2A^pro^] the GFP transgene with the mutated 2A^pro^-CS from the pJet-PD-VP1-GFP-2A construct was amplified and assembled using the In-Fusion HD Cloning Kit (Takara Bio). For the construct pJet-PD-GFP-VP4 [3C^pro^] the 2A^pro^-CS of pJet-PD-GFP-VP4 [2A^pro^] was modified by site-directed mutagenesis using mutagenic primers to generate an artificial 3C^pro^-CS. All primers were designed using the Infusion Primer Design Tool (Takara Bio).

### Cloning of transgenes into the cDNA of PD-H

For the constructs pJet-PD-90, pJet-PD-180, pJet-PD-357, and pJet-PD-534, which contain inserts smaller than the coding sequence of GFP, the plasmid pJet-PD-VP1-GFP-2A served as the template. The transgene length was reduced to 90, 180, 357, or 534 bp by truncating the GFP coding region at the 3′ end. Specific primers were designed using the In-Fusion Primer Design Tool (Takara Bio), and the constructs were assembled with the In-Fusion HD Cloning Kit (Takara Bio). The constructs pJet-PD-894, pJet-PD-1071, and pJet-PD-1428 contain foreign inserts of 894, 1,071, and 1,428 bp, respectively. In these cases, pJet-PD-VP1-GFP-2A was also used as the template plasmid. The GFP coding sequence was replaced with firefly luciferase fragments of the indicated lengths, derived from the psi-CHECK3 plasmid (Addgene, Watertown, MA, USA). The luciferase sequence was truncated at the 3' end to generate inserts of the desired size. The cDNA of Neo-2/15 was a kind gift from David Baker (Department of Biochemistry, University of Washington, Seattle, WA, USA). Initially, the cDNA was cloned into a pUC19 vector using specific primers designed with the In-Fusion Primer Design Tool (Takara Bio), and the construct was assembled with the In-Fusion HD Cloning Kit. To generate the plasmid pJet-PD-Neo-2/15, the GFP sequence in pJet-PD-VP1-GFP-2A was replaced with the Neo-2/15 sequence from the pUC19 vector, using specific primers and the In-Fusion HD Cloning Kit. The plasmid pJet-PD-Neo-2/15-His was derived from pJet-PD-Neo-2/15 by adding a C-terminal His-tag, using the In-Fusion HD Cloning Kit. Finally, the plasmid pJet-PD-GFP_trunc_ was created by truncating the GFP coding sequence at the 3′ end in pJet-PD-VP1-GFP-2A, yielding a GFP fragment of the exact same size as Neo-2/15.

### Generation of viruses

For generation of recombinant viruses, HEK293T cells were seeded into 6-well plates and transfected at a confluence of 70%–80% with 2.5 μg of the corresponding plasmid containing viral cDNA using PEImax (Polysciences Europe GmbH, Hirschberg an der Bergstraβe, Germany). Cells were disrupted 72 h after transfection by three freeze-thaw-cycles. Cell debris was removed, and the supernatant was used for determination of virus titers. GFP expression was observed 48 and 72 h after transfection with a fluorescence microscope (Observer Z1, Carl Zeiss, Oberkochen Germany) from viruses containing a GFP transgene. To obtain higher titer, all viruses were amplified in HEK293T cells. For *in vivo* experiments viruses were concentrated and purified using a sucrose gradient as described previously.^49^

### Sequencing of viral genome

To sequence the inserted transgenes, viral RNA was extracted using the NucleoSpin RNA Virus Kit (Macherey-Nagel, Düren, Germany) following the manufacturer’s protocol. Reverse transcription was performed with the High-Capacity cDNA Reverse Transcription Kit (Applied Biosystems, Foster City, CA, USA). PCR fragments were generated using CloneAmp HiFi PCR Premix (Takara Bio) and CVB3-specific primers flanking the transgene region. Sanger sequencing was carried out by LGC Biosearch Technologies (Berlin, Germany). Sequence alignment was performed with SnapGene 5.1.7 software (SnapGene, San Diego, CA, USA).

### Virus growth curves

Virus replication was analyzed using viral growth curves. Specifically, 4 × 10^4^ HEK293T cells, 2 × 10^4^ HeLa cells, 5 × 10^4^ Colon-26 cells, or 2 × 10^4^ CaCo-2 cells were seeded into 96-well plates. After 24 h, the medium was removed, and the cells were infected with 100 μL of a virus suspension at a MOI of 0.1. Following 1 h incubation, the medium was replaced with 200 μL of fresh medium. At 0, 8, 24, and 48 h post-infection, the cells underwent three freeze-thaw cycles. Cell debris was then removed by centrifugation, and the supernatant was collected for virus titer determination.

### Cell viability assay

Cell viability was assessed using the Cell Proliferation Kit (XTT) (Promega GmbH, Walldorf, Germany) according to the manufacturer’s instructions. In brief, 4 × 10^4^ HEK293T cells, 2 × 10^4^ HeLa cells, 5 × 10^4^ Colon-26 cells, or 2 × 10^4^ CaCo-2 cells were seeded into 96-well plates. The following day, when the cells reached approximately 80% confluence, cells were infected with virus. Absorbance was measured at 24 and 48 h post-infection using the TriStar2 LB 942 Multimode Microplate Reader (Berthold Technologies, Bad Wildbad, Germany). Cells treated with 50 μL of 5% Triton X-100 solution served as a control.

### Determination of virus titer

For determination of virus titer, 1.5 × 10^4^ HeLa cells were seeded in 96-well plates. After 24 h, 80 μL of a 10-fold serial dilution of the virus samples in MEM medium (Gibco) were added to the cells. Plates were then incubated for 72 h and observed by an inverted microscope. If cells had developed a cytopathic effect (CPE) compared to the uninfected controls, the well was marked as positive. Wells without CPE were marked as negative. The calculation of TCID_50_ titers was done with Sparemann & Kärber algorithm.

### Serial passaging of PD-H variants

For serial passaging 1 × 10^6^ HeLa cells, 1.5 × 10^6^ Colon-26 cells, or 1 × 10^6^ CaCo-2 cells per well were seeded in 6-well plates. After 24 h, the cells were infected with MOI 0.1 of CVB3 PD-90, PD-180, PD-357, PD-534, PD-GFP, and PD-1071 for passage 1. The cells were incubated with the virus solution for 48 h. The viruses were isolated by three freeze-thaw cycles, cell debris was removed by centrifugation and virus titer was determined as described. This process was repeated until 10 virus passages were performed. Viruses were stored at −20°C until use.

### Assessment of transgene stability

To assess, until which passage the inserted transgene remains stable, viral RNA of each passage was extracted with a NucleoSpin RNA Virus Kit (Macherey-Nagel) according to the manufacturer’s instructions. Reverse transcription was carried out with the High-Capacity cDNA Reverse Transcription Kit (Applied Biosystems). For the generation of PCR fragments, CloneAmp HiFi PCR Premix (Takara Bio) with CVB3-specific primers flanking the transgene region was used. PCR products were analyzed on a 1% agarose gel.

### IL-2Rβγ bioassay

The functionality of the viral expressed IL-2 derivative Neo-2/15 and Neo-2/15-His was investigated by IL-2Rβγ bioassay (Promega GmbH, Walldorf, Germany) according to the manufacturer’s instructions. Therefore, 1 × 10^6^ HEK293T cells were seeded in 6-well plates and infected 24 h later with PD-H, PD-Neo-2/15 and PD-Neo-2/15-His at MOI 0.1 and 0.01. Forty-eight h after infection, the supernatant of infected cells was collected, centrifuged to remove cell debris and 25 μL of the supernatant was used in the IL-2Rβγ bioassay. To inactivate the virus before analysis with the bioassay, samples were incubated for 30 min at 60°C in a thermoblock.

### Isolation and purification of CD3^+^ T cells from peripheral blood

Peripheral blood mononuclear cells (PBMCs) were isolated from fresh whole blood by density gradient centrifugation using Ficoll (Bio&SELL, Feucht, Germany). CD3^+^ T cells were then enriched via magnetic-activated cell sorting (MACS). Briefly, PBMCs were incubated with anti-CD3 microbeads (Miltenyi Biotec, Bergisch Gladbach, Germany) for 30 min at 4°C, applied to an LS column (Miltenyi Biotec) in a magnetic field. The CD3^−^ flow-through was collected and the CD3^+^ cells were eluted after removing the column from the magnet. To assess purity, both CD3^+^ and CD3^−^ fractions were stained with anti-CD3-FITC antibodies (Miltenyi Biotec) for 20 min at room temperature in the dark, washed twice with PBS containing BSA and EDTA, and resuspended in 200 μL of the same buffer. Samples were analyzed using a NorthernLights spectral flow cytometer (Cytek Biosciences, Fremont, CA, USA). The CD3^+^ fraction showed 96% purity. Cells were cryopreserved in freezing medium (10% DMSO in FCS) and stored at −80°C until use.

### Stimulation of primary human T cells

To evaluate whether virus-expressed Neo-2/15 induces proliferation of primary human T cells, 1 × 10^7^ cryopreserved CD3^+^ T cells were thawed in RPMI-1640 supplemented with 2% FCS and 1:10,000 Benzonase (Sigma-Aldrich, St. Louis, MO, USA). To stimulate the cells, 48-well plates were pre-coated with anti-CD3 and anti-CD28 antibodies (both BD Biosciences, San Jose, CA, USA) for 2 h at 37°C and 1 × 10^6^ T cells per well were seeded in RPMI-1640 containing P/S (Gibco) and human AB serum (Sigma-Aldrich). The cells were thereafter treated with either 3 ng/mL recombinant human IL-2 (Miltenyi Biotec) or 50 μL heat-inactivated Neo-2/15-containing supernatant from HEK293T cells infected at MOI 0.1 for 48 h. All cultures were adjusted to 1 mL final volume and incubated for 48 h at 37°C. Afterward, cells were gently resuspended, washed with PBS, and counted using a CASY cell counter (Roche Innovatis AG, Bielefeld, Germany). Unstimulated (freshly thawed) CD3^+^ T cells were processed in parallel to determine baseline cell numbers.

### Determination of T cell proliferation

To assess T cell proliferation, cells were resuspended in 500 μL PBS and mixed with 500 μL PBS containing 0.25 μM CFDA SE (Sigma-Aldrich). After 4 min incubation at room temperature, the reaction was stopped by adding 2 mL PBS with 0.5% BSA. Cells were washed twice with PBS/BSA to remove excess dye, then resuspended in RPMI with 1% P/S and 10% AB serum. Cells were seeded in 96-well plates at 3 × 10^4^ cells/well and cultured for 5 days at 37°C, 5% CO_2_ with either no additives, 12 ng/mL recombinant human IL-2 (Miltenyi Biotec), or heat-inactivated viral supernatant containing Neo-2/15 at final volumes of 1, 2, 5, 10, 25, or 50 μL per well. After 5 days, cells were harvested, washed twice with PBS/0.5% BSA, and stained with Zombie Yellow viability dye (BioLegend) for 10 min at room temperature in the dark. Surface staining was performed using anti-CD4 VioBright V600 (Miltenyi Biotec) and anti-CD8 VioBlue antibodies (Miltenyi Biotec) for 20 min at room temperature in the dark. Cells were washed with PBS containing BSA and EDTA, resuspended in 200 μL buffer, and analyzed on an Aurora spectral flow cytometer (Cytek Biosciences). Data were processed with FlowJo v.10.6.2 (BD Biosciences).

### Syngenic s.c. Colon-26 *in vivo* model

For generation of Colon-26 tumors, 6-week-old female Balb/C mice (Charles River Laboratories, Sulzfeld, Germany) were injected with 5 × 10^5^ cells s.c. into the right flank. After 5 days, when tumor volume reached ∼25 mm^3^, the tumors were injected with 5 × 10^6^ PFU of PD-H, PD-Neo-2/15, or PD-GFP_trunc_ in 30 μL PBS. Control animals were injected with 30 μL PBS. The injections were repeated on day 6 and 7. Tumor volume was measured every 2 days. Animals were sacrificed at day 10 after virus injection and analyzed. For analysis of transgene stability, tumors were injected with 5 × 10^6^ PFU of PD-Neo-2/15 5 days after tumor establishment. Animals were sacrificed 3 days after virus injection. The animal experiments were performed in accordance with the principles of laboratory animal care and all German laws regarding animal protection and approved by the responsible local authorities (State Office of Health and Social Affairs, Berlin, Germany, reference number G 0073/23).

### Histopathological analysis

Tissues were fixed in 4% paraformaldehyde and embedded in paraffin. Five-micrometer-thick sections were prepared and stained with hematoxylin and eosin (H&E) and analyzed for tissue destruction and inflammation.

### Quantitative determination of CVB3 RNA levels by RT-qPCR

Total RNA was extracted from pancreatic tissue using TRIzol Reagent (Thermo Fisher Scientific) following the manufacturer’s protocol. Tissue homogenization was carried out with Pellet Pestles. The isolated RNA was stored at −80°C until further processing. cDNA was synthesized from viral RNA using Multiscribe Reverse Transcriptase (Applied Biosystems, Foster City, CA, USA). Quantification of CVB3 RNA was subsequently performed via qPCR employing the SsoFast EvaGreen Supermix (Bio-Rad Laboratories, Hercules, CA, USA) together with CVB3-specific primers. RNA copy numbers were calculated for each sample based on the Ct values obtained from a CVB3 standard curve.

### MNC isolation from tumors and flow cytometry analysis

Tumor mononuclear cells (MNCs) were isolated from Colon-26 tumors 10 days after first i.t. injection using the Neonatal Heart Dissociation Kit (Miltenyi Biotec, Bergisch Gladbach, Germany) and gentle MACS Octo Dissociator (Miltenyi Biotec), according to the manufacturer’s instructions.

Flow cytometry analysis of tumor MNCs was performed using directly conjugated monoclonal mouse antibodies: CD4 FITC (BioLegend, San Diego, CA, USA), CD8a APC Cy7 (BD Biosciences, Franklin Lakes, NJ, USA), CD11b PE/Cy7 (BioLegend), CD49 PerCP/Cy5.5 (BioLegend), and F4/80+ PacBlue (BioLegend). Surface staining was performed according to the manufacturer’s instructions. Sample analysis was performed on a MACSQuant Analyzer (MACSQuant10, Miltenyi Biotec) and flow cytometry data were analyzed with FlowJo 8.7. software (FlowJo, LLC, New York, NY, USA).

### Statistical analysis

Statistical analysis was conducted using GraphPad Prism 8.2 software (GraphPad Software, Boston, MA, USA). A two-tailed unpaired Student’s *t* test was employed to assess statistical significance. For the analysis of the animal experiments, the Mann-Whitney *U* test was used to assess statistical significance. Differences were deemed statistically significant at *p* < 0.05.

## Data and code availability

Any materials and data described in this study will be made available in a timely fashion to members of the scientific community for non-commercial purposes.

## Acknowledgments

This project was funded by 10.13039/501100005972Deutsche Krebshilfe (grants 70115068 to H.F. and 70115119 to S.V.L.), the 10.13039/501100001659Deutsche Forschungsgemeinschaft (grant 536819681 to H.F., S.V.L., and R.K.), the 10.13039/100008672Wilhelm Sander-Stiftung (grant 2024.127.1 to H.F.), and internal funding from TU Berlin’s ProTuTec program (20016/TUB).

The use of human-derived material was conducted in accordance with the Declaration of Helsinki and approved by the Ethics Committee of Charité, Universitätsmedizin Berlin (approval number EA2/067/15). All animal experiments were performed in accordance with institutional and national guidelines for animal welfare and were approved by the State Office of Health and Social Affairs (Landesamt für Gesundheit und Soziales, LaGeSo), Berlin, Germany (approval number G0073/23). Graphical abstract was created with BioRender.com.

## Author contributions

Methodology, A.G., B.D., L.E., A.H., and L.H.; investigation, B.D., L.E., M.G., J.S.-M., and R.K.; formal analysis, L.E.; resources and supervision, A.T., H.F., J.K., R.K., and S.V.L.; conceptualization, funding acquisition, and project administration, H.F., S.V.L.; validation; writing – original draft, H.F. and L.E.; writing – review and editing, A.G., A.T., H.F., J.K., R.K., and S.V.L.

## Declaration of interests

H.F., A.H., L.E., A.G., and B.D. have a patent pending for transgene expressing CVB3.

## Declaration of generative AI and AI-assisted technologies in the writing process

During the preparation of this manuscript, the authors used ChatGPT (OpenAI) for linguistic support and improvement of clarity. The authors subsequently reviewed and revised the manuscript and assume full responsibility for its content.
